# GPR120 promotes neutrophil control of intestinal bacterial infection

**DOI:** 10.1080/19490976.2023.2190311

**Published:** 2023-03-16

**Authors:** Zheng Zhou, Wenjing Yang, Tianming Yu, Yu Yu, Xiaojing Zhao, Yanbo Yu, Chuncai Gu, Anthony J Bilotta, Suxia Yao, Qihong Zhao, George Golovko, Mingsong Li, Yingzi Cong

**Affiliations:** a Department of Microbiology and Immunology, University of Texas Medical Branch, Galveston, USA; b Department of Gastroenterology, Nan Fang Hospital, Southern Medical University, Guangzhou, China; c Department of Gastroenterology, Third Affiliated Hospital of Guangzhou Medical University, Guangzhou, China; d Bristol-MyersSquibb, Princeton, New Jersey, USA; e Department of Pharmacology and Toxicology, University of Texas Medical Branch, Galveston, USA; f Department of Pathology, University of Texas Medical Branch, Galveston, USA

**Keywords:** Neutrophil, GPR120, gut microbiota, Interic infection, Intestinal inflammation

## Abstract

G-protein coupled receptor 120 (GPR 120) has been implicated in anti-inflammatory functions. However, how GPR120 regulates the neutrophil function remains unknown. This study investigated the role of GPR120 in the regulation of neutrophil function against enteric bacteria. 16S rRNA sequencing was used for measuring the gut microbiota of wild-type (WT) mice and *Gpr120*^−/−^ mice. *Citrobacter rodentium* infection and dextran sulfate sodium (DSS)-induced colitis models were performed in WT and *Gpr120*^−/−^ mice. Mouse peritoneal-derived primary neutrophils were used to determine the neutrophil functions. *Gpr120*^−/−^ mice showed altered microbiota composition. *Gpr120*^−/−^ mice exhibited less capacity to clear intestinal *Citrobacter rodentium* and more severe intestinal inflammation upon infection or DSS insults. Depletion of neutrophils decreased the intestinal clearance of *Citrobacter rodentium*. GPR120 agonist, CpdA, enhanced WT neutrophil production of reactive oxygen species (ROS) and extracellular traps (NETs), and GPR120-deficient neutrophils demonstrated a lower level of ROS and NETs. CpdA-treated neutrophils showed an enhanced capacity to inhibit the growth of *Citrobacter rodentium*, which was abrogated by the inhibition of either NETs or ROS. CpdA promoted neutrophil inhibition of the growth of commensal bacteria *Escherichia coli* O9:H4 and pathobiont *Escherichia coli* O83:H1 isolated from a Crohn’s disease patient. Mechanically, mTOR activation and glycolysis mediated GPR120 induction of ROS and NETs in neutrophils. Additionally, CpdA promoted the neutrophil production of IL-17 and IL-22, and treatment with a conditioned medium of GPR120-activated neutrophils increased intestinal epithelial cell barrier functions. Our study demonstrated the critical role of GPR120 in neutrophils in protection against enteric bacterial invasion.

## Introduction

The intestinal mucosa consists of a single layer of epithelial cells covered by a mucus layer and a number of immune cells underneath. Under normal steady conditions, the intestine is separated from a large quantity of the microbiome, dietary substance, and ingested toxins by the mucus layer and epithelial cells. Meanwhile, there are a lot of immune cells that accumulate into injured mucosa, where commensal microbiota and pathogens invade when intestinal inflammation occurs^1^. It has been shown that innate immune cells, especially neutrophils, are crucial to protecting against bacterial invasion in the intestine^2,3^.

Neutrophils have long been viewed as the effector cells in acute and chronic inflammation^4^. Large numbers of neutrophils accumulate in the intestinal mucosa and phagocytose pathogenic microbes upon intestinal inflammation, which damages the intestinal barrier^5^. However, the protective role of neutrophils has also been recognized in regulating intestinal inflammation^3^. It has been reported that neutrophils can eliminate bacteria through the production of reactive oxygen species (ROS), formation of extracellular traps (NETs), and secretion of several cytokines^6–8^. However, how the neutrophil function is regulated is still not completely understood.

Dietary ω-3 polyunsaturated fatty acids (PUFA) have been implicated in regulating intestinal diseases^9^. As a receptor for ω-3 PUFA, G-protein coupled receptor 120 (GPR120) plays a critical role in various physiologic homeostasis mechanisms^10^. It has also been shown that GPR120 agonist inhibits proinflammatory cytokine production by macrophages and promotes IL-10 production in CD4^+^ T cells^11–13^. Although its role in adipocytes, obesity, and diabetes is well established^11,14,15^, the effect of GPR120 in regulation of intestinal microbiota homeostasis is still unknown. In this report, we demonstrated that GPR120 enhances neutrophil function in controlling gut bacteria, which contributes to inhibiting intestinal inflammation and infection. GPR120 agonist promotes neutrophils to inhibit bacterial growth through upregulation of ROS production and NETs formation, which is mediated by mTOR and glycolysis. In addition, GPR120 increases the neutrophil production of IL-17A and IL-22 and intestinal epithelial cell barrier function.

## Results

### GPR 120 regulates the gut microbiota.

**Figure 1.**
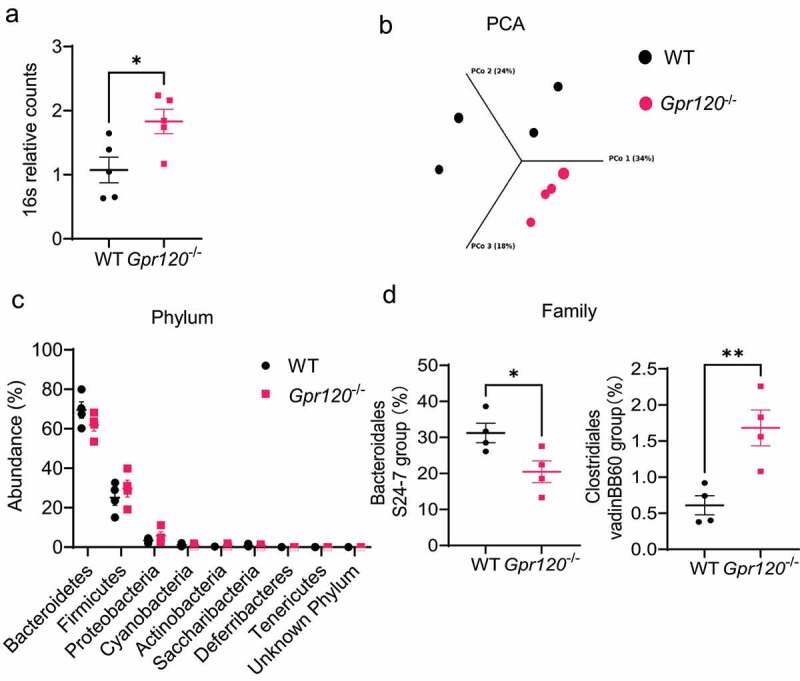
*Gpr120*^−/−^ mice demonstrate altered gut microbiota composition.

Notes: (**a**) 16S counts were measured in fecal pellets collected from WT and *Gpr120*^−/−^ mice using qRT-PCR. (**b-d**) Fecal pellets were collected from WT and *Gpr120*^−/−^ mice, and 16s rRNA sequencing was performed. PCA analyses were determined using Bray-Curtis comparison (**b**). The differences in microbiota abundance by phylum were determined between WT and *Gpr120*^−/−^ mice (**c**). The differences in the *Bacteroidales* S24-7 group and *Clostridiales* vadinBB60 group were determined between WT and *Gpr120*^−/−^ mice (**d**). Data were expressed as mean ± SEM. Statistical significance was tested by a two-tailed unpaired Student t-test (**a, c**, and **d**) or Bray-Curtis comparison (**b**). *p < 0.05, **p < 0.01.

To determine the role of GPR 120 in regulating gut microbiota, we assessed the total gut microbiota between WT and *Gpr* 120^−/−^ mice by determining 16S DNA counts in feces. We found that there were more bacteria in *Gpr* 120^−/−^ mice compared with WT mice (Figure 1a). We then determined the intestinal microbiota composition using 16S rRNA sequencing analysis. The principal coordinates based on a Bray-Curtis comparison clearly separated the samples from the WT and *Gpr* 120^−/−^ mice (Figure 1b). Taxonomically, although there were no significant differences in relative bacteria abundance at the phylum level (Figure 1c), *Gpr120*^−/−^ mice exhibited a decreased tendency to harbor *Bacteroidetes*, which has been shown to be decreased in patients with inflammatory bowel disease (IBD)^16^. Furthermore, we found that *Bacteroidales* S24–7 group, which belongs to *Bacteroidetes* phylum, was significantly decreased, and *Clostridiales* vadinBB60 group, which belongs to *Firmicutes phylum*, was increased (Figure 1d). Taken together, these results indicate that GPR120 regulates the growth of certain gut microbiota.

### Deficiency of GPR120 promotes intestinal inflammation with decreasing clearance of intestinal pathogen

**Figure 2.**
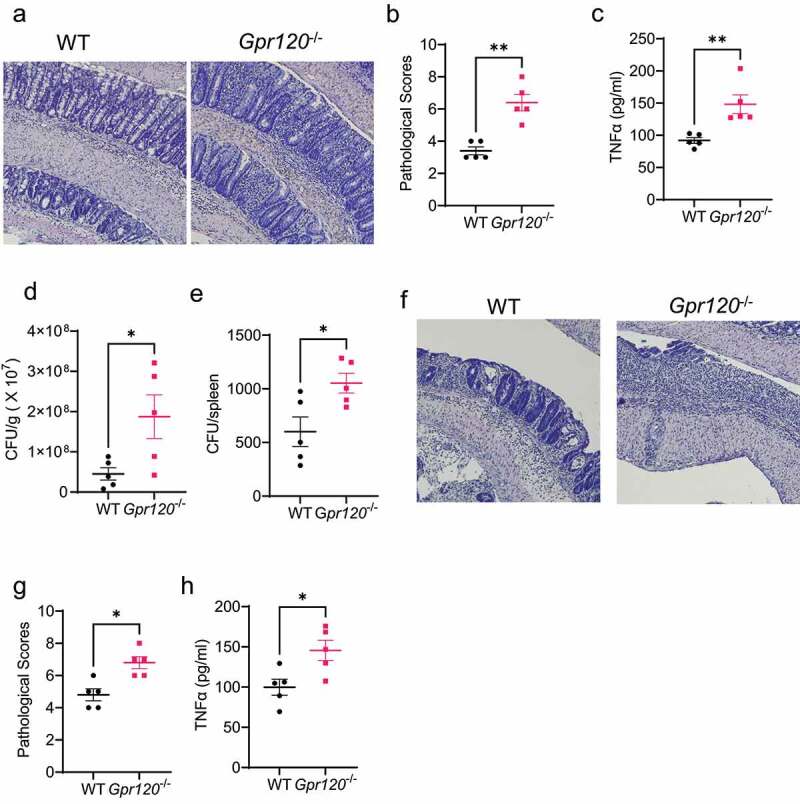
*Gpr120*^−/−^ mice are impaired in the clearance of intestinal *Citrobacter rodentium* and are susceptible to intestinal inflammation.

Notes: (**a-e**) WT and *Gpr120*^−/−^ mice (n = 5) were infected with *Citrobacter rodentium*(5 × 10^8^ colony‐forming units (CFU)/mouse) by oral gavage. Mice were sacrificed on day 10 post-infection. (**a**) Representative histopathology images of the colons were shown. (**b**) Histological scores were assessed. (**c**) TNF-α secretion in colons was determined. (**d-e**) Fecal pallets and spleens were collected on day 10, and CFU in feces (**d**) and spleens (**e**) were quantified. (**f-h**) WT and *Gpr120*^−/−^ mice (n = 5) were treated with 2% DSS in drinking water for 7 days and normal drinking water for another 3 days. Mice were sacrificed on day 10. (**f**) Representative histopathology images of the colons were shown. (**g**) Histological scores were assessed. (**h**) TNF-α secretion in colons was determined. One representative of three independent experiments was shown. Data were expressed as mean ± SEM. Statistical significance was tested by the non-parametric two-tailed Mann-Whitney U test (**b and g**) or the two-tailed unpaired Student's t-test (**c-e, and h**). *p < 0.05, **p < 0.01.

To investigate whether GPR120 regulates pathogen clearance and intestinal inflammation, we first performed *Citrobacter rodentium*, which is similar to human enteropathogenic Escherichia coli associated with IBD [16], infection in WT and *Gpr120*^−/−^ mice. After 10 days, we found that *Gpr120*^−/−^ mice demonstrated more severe colitis compared to WT mice, as demonstrated by higher pathological scores and elevated intestinal TNF-α expression (Figures 2a-c). In addition, *Gpr120*^−/−^ mice exhibited increased intestinal *Citrobacter rodentium* counts in feces and spleens (Figures 2d-e), indicating that deficiency of GPR120 decreases intestinal pathogen clearance and promotes bacteria translocation to the spleen. To confirm the role of GPR120 in regulating intestinal inflammation, we performed DSS-induced colitis in WT and *Gpr120*^−/−^ mice. Consistently, deficiency of GPR120 exacerbated intestinal inflammation induced by DSS (Figures 2f-h). These data suggested the importance of GPR120 in the clearance of intestinal pathogens and control of intestinal inflammation.

### Neutrophils protect the intestine against enteric infection of Citrobacter rodentium

**Figure 3.**
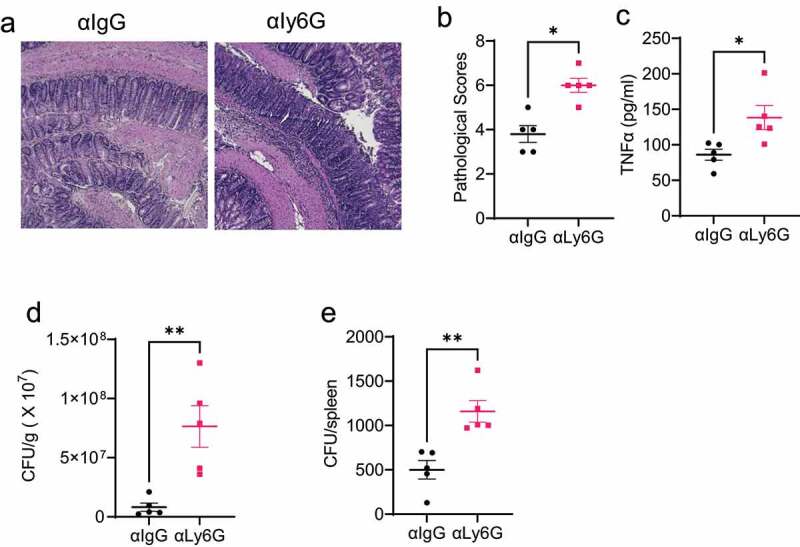
Depletion of neutrophils decreases the intestinal clearance of *Citrobacter rodentium*.

Notes: WT mice (n = 5/group) were infected with *Citrobacter rodentium*(5 × 10^8^ colony‐forming units (CFU)/mouse) by oral gavage. One group of mice was administered with anti-Ly6G antibody, and another was given anti-IgG antibody as a control daily. Mice were sacrificed on day 10 post-infection. (**a**) Representative histopathology images of the colons were shown. (**b**) Histological scores were assessed. (**c**) TNF-α secretion in colons was determined. (**d-e**) Fecal pallets and spleens were collected on day 10, and CFU in feces (**d**) and spleens (**e**) were quantified. One representative of three independent experiments was shown. Data were expressed as mean ± SEM. Statistical significance was tested by the non-parametric two-tailed Mann-Whitney U test (**b**) or the two-tailed unpaired Student t-test (**c-e**). *p < 0.05, **p < 0.01.

As the first line of defense against gut infection, massive neutrophils accumulate at the inflammation site. We next investigated the role of neutrophils in protecting the host against enteric infection. The WT mice were orally infected with *Citrobacter rodentium*, and then treated with the control IgG antibody or anti-Ly-6 G neutralizing antibody to deplete neutrophils. Mouse weights were monitored daily, and mice were sacrificed on day 10. The efficiency of the depletion was shown in Supplementary Figure S1A-C. Neutrophil-depleted mice demonstrated more severe intestinal inflammation (Figures 3a-b) than the IgG-treated mice. Additionally, intestinal TNFα secretion was increased in the neutrophil-depleted mice (Figure 3c). Furthermore, the depletion of neutrophils increased *Citrobacter rodentium* counts in feces (Figure 3d) and spleens (Figure 3e), indicating that neutrophils enhance the clearance of *Citrobacter rodentium* in the intestine and decrease bacteria translocation to other organs.

### GPR120 enhances neutrophil functions related to bacterial killing

**Figure 4.**
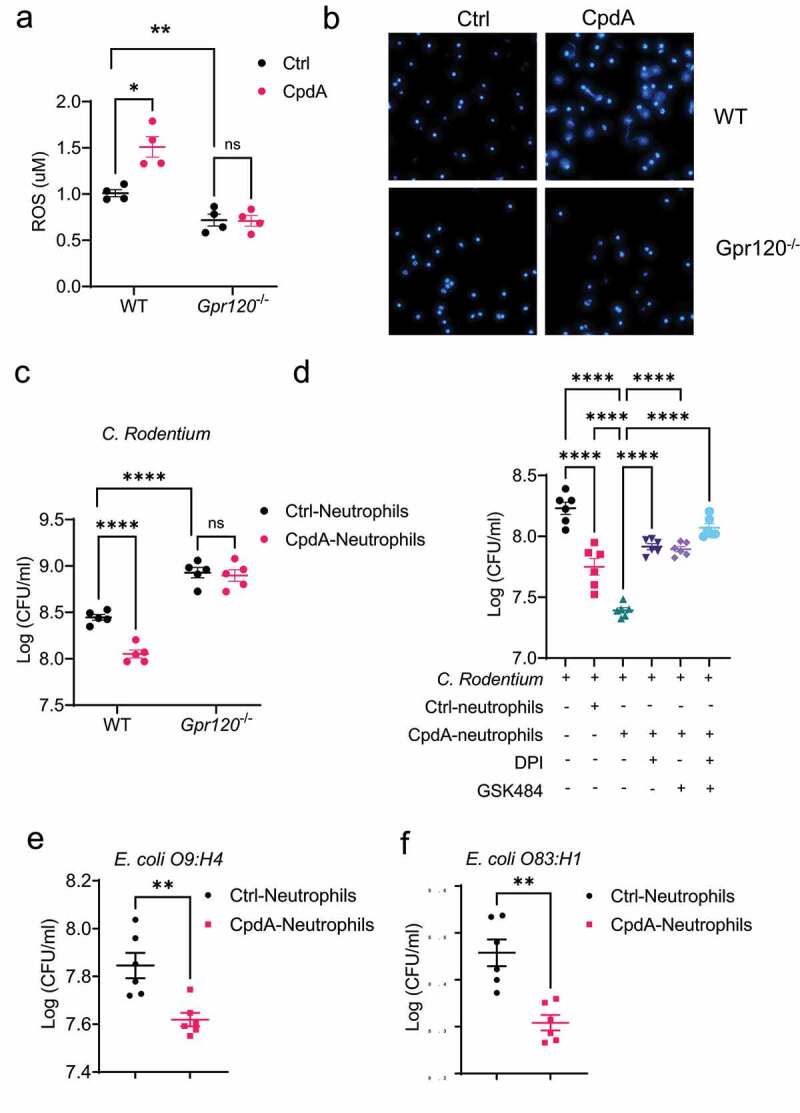
GPR120 agonist promotes neutrophil inhibition of enteric bacterial growth through the upregulation of ROS and NETs.

Notes: (**a-b**) WT or GPR120-deficient neutrophils (n = 4/group) were treated with or without CpdA (3 µM) for 1 h. ROS production was measured using the Amplex Red Hydrogen Peroxide Assay Kit (**a**). WT or GPR120-deficient neutrophils were then stained with Hoechst 33342 (blue), and representative NETs were shown (**b**). (**c**) WT or GPR120-deficient neutrophils (n = 5/group) were pre-treated with or without CpdA (3 µM) for 1 h, and then co-cultured with *Citrobacter rodentium* in the plates for 12 h. The bacterial suspensions were then transferred to solid MacConkey’s agar culture plates overnight, and CFU was counted. (**d**) WT neutrophils (n = 6/group) were pre-treated with or without CpdA (3 µM) for 1 h and then co-cultured with *Citrobacter rodentium* (or *Citrobacter rodentium* were cultured alone) in the presence of DPI or/and GSK484 in the plates for 12 h. The bacterial suspensions were then transferred to solid MacConkey’s agar culture plates overnight, and CFU was counted in the plates for 12 h. The bacterial suspensions were then transferred to solid MacConkey’s agar culture plates overnight, and CFU was counted. (**e-f**) Neutrophils were pre-treated with or without CpdA (3 µM) for 1 h, and then co-cultured with *Escherichia coli* O9:H4 (**e**) and *Escherichia coli* O83:H1 (**f**) for 12 h. The bacterial suspensions were then transferred to Luria Broth’s agar culture plates overnight, and CFU was counted. One representative of three independent experiments was shown. Data were expressed as mean ± SEM. Statistical significance was tested by the two-tailed unpaired Student’s t-test (**a**, **c**, and **e-f**) or one-way ANOVA (**d**). **p < 0.01, ***p < 0.001, ***p < 0.0001.

Given that GPR120 regulates intestinal inflammation and intestinal bacteria clearance (Figure 2), and neutrophils are crucial for protection from enteric pathogen infection (Figure 3), we next asked whether GPR120 modulates neutrophil functions to clear bacteria. Neutrophils are involved in killing bacteria and pathogens, which is mainly dependent on the production of ROS and the formation of extracellular traps (NETs)^17–20^. Because of the short life of freshly prepared intestinal neutrophils and the easy preparation of peritoneal neutrophils in large quantities^21^, we used peritoneal neutrophils for *in vitro* experiments in this study. The purity of prepared peritoneal neutrophils was regularly >95% (Supplementary Figure S2A). We treated WT and GPR120-deficient neutrophils with or without CpdA, a GPR120-selective agonist^22^. We first determined the toxicity of CpdA on neutrophils and found that CpdA did not affect neutrophils’ viability with doses less than 10 µM (Supplementary Figures S2B-D). Next, we determined whether GPR120 affects ROS production in neutrophils. CpdA-treated WT, but not GPR120-deficient, neutrophils produced a higher level of ROS than control neutrophils (Figure 4a), indicating that CpdA specifically affects on GPR120. In addition, the ROS level was decreased in GPR120-deficient neutrophils compared with WT neutrophils. Then, we investigated the role of GPR120 in regulating the formation of NETs. Neutrophils were treated with or without CpdA in the presence of Hoechst 33,342, a dye that stains the primary component of NETs and nucleic acid. We found that CpdA promoted NETs formation in WT neutrophils, and the level of NETs was decreased in GPR120-deficient neutrophils compared with WT neutrophils (Figure 4b). Consistently, treatment with DHA, a ω-3 PUFA, a natural GPR 120 ligand, promoted ROS production and NETs formation (Supplementary Figures S3A-B).

### CpdA promotes the neutrophil killing of bacteria

To investigate whether CpdA affects neutrophils to kill bacteria, we conducted an anti-bacterial experiment by culturing *Citrobacter rodentium* with CpdA-treated or control WT and GPR120-deficient neutrophils. As shown in Figure 4c, CpdA-treated WT, but not GPR120-deficient, neutrophils significantly reduced *Citrobacter rodentium* counts compared with control neutrophils, while the counts were higher when cultured with GPR120-deficient neutrophils compared with WT neutrophils. In addition, DHA-pre-treated neutrophils showed higher capacity to inhibit the growth of *Citrobacter rodentium* (Supplementary Figure S3C). To determine whether GPR120 promotes neutrophils to kill bacteria through the induction of ROS or/and NETs formation, we cultured *Citrobacter rodentium* with CpdA-treated or control neutrophils in the presence of the ROS inhibitor, Diphenyleneiodonium (DPI)^23^, or the NETs inhibitor, GSK484^19^. Both control and CpdA-treated neutrophils suppressed the growth of *Citrobacter rodentium* (Figure 4d). Inhibition of ROS or NETs abrogated the CpdA-neutrophil inhibition of bacterial growth (Figure 4d), which was enhanced by the combination of these two inhibitors. In addition, DPI and GSK484 themselves had no effect on growth of *Citrobacter rodentium* (Supplementary Figure S4). These data indicated that CpdA enhances neutrophil killing of bacteria at least partially through induction of ROS and NETs.

Next, we investigated whether GPR120 enhancement of neutrophil killing is bacterial strain-specific, we cultured *Escherichia coli* O9:H4, the intestinal commensal bacteria, with CpdA-treated or control neutrophils. CpdA promoted the neutrophil killing of the commensal bacteria (Figure 4e). To determine whether GPR120 also enhances neutrophil killing of pathogenic bacteria in IBD, we cultured *Escherichia coli* O83:H1, a pathobiont isolated from a patient with Crohn’s disease^24^, with CpdA-treated or control neutrophils. We found that CpdA-treated neutrophils inhibited the growth of *Escherichia coli* O83:H1 (Figure 4f). Taken all together, these data demonstrated that GPR120 enhances neutrophil killing of both gut commensal bacteria and pathobionts.

### GPR120 promotes the neutrophil killing of bacteria through the activation of the mTOR pathway

**Figure 5.**
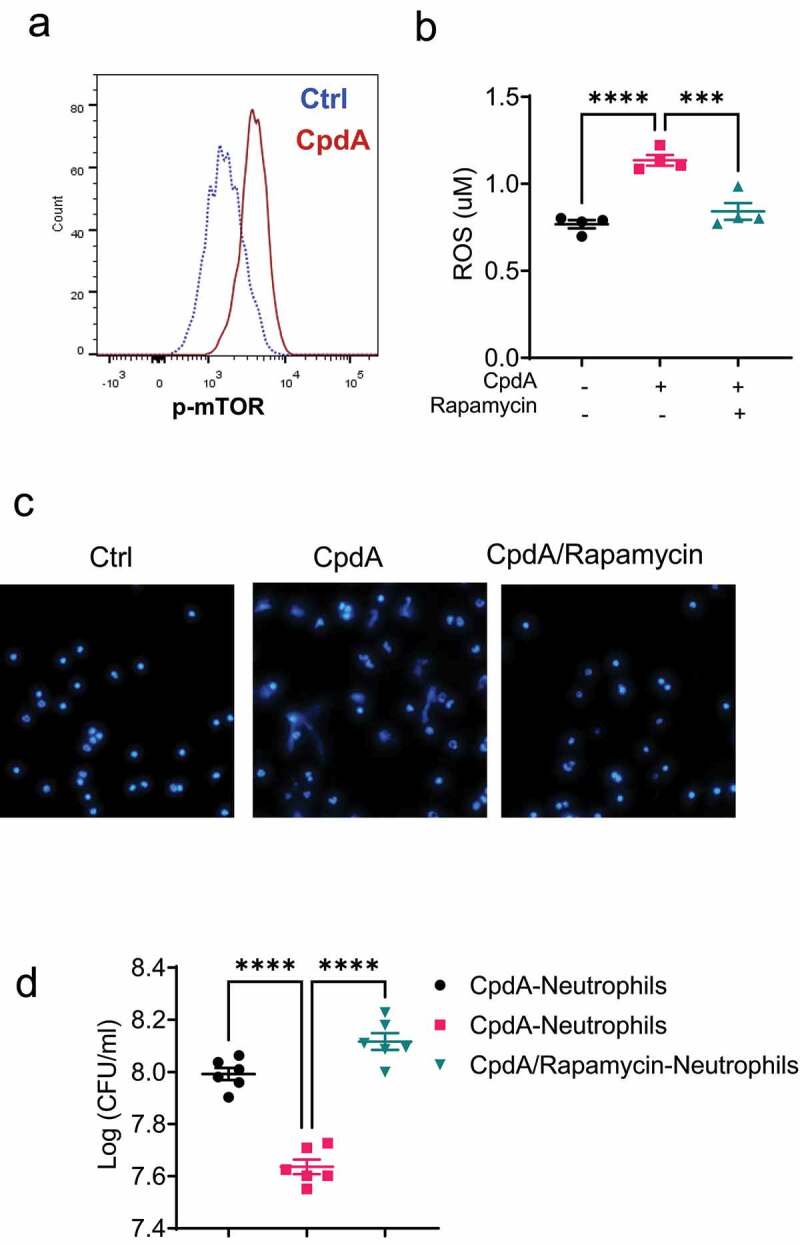
mTOR mediates GPR120 induction of neutrophil production of ROS and formation of NETs.

Notes: (**a**) Neutrophils were treated with or without CpdA (3 µM) for 5 min. The phosphorylated mTOR levels were determined by FACS. (**b-c**) Neutrophils were treated with or without CpdA (3 µM) in the presence or absence of rapamycin (2 μM) for 1 h. ROS production (**b**) and NETs formation (**c**) were assessed. (**d**) Neutrophils were pre-treated with or without CpdA (3 µM) in the presence or absence of rapamycin (2 μM) for 1 h, and then co-cultured with *Citrobacter rodentium* in the plates for 12 h. The bacterial suspensions were then transferred to solid MacConkey’s agar culture plates overnight, and CFU was counted. One representative of three independent experiments was shown. Data were expressed as mean ± SEM. Statistical significance was tested using the two-tailed one-way ANOVA (**b** and **d**). ***p < 0.001, ***p < 0.0001.

To investigate the mechanisms underlying GPR120 regulation of neutrophil functions, we determined whether GPR120 enhances mTOR activation, which has been reported to mediate several functions regulated by GPR120 in other cell types^25^. We found that CpdA promoted mTOR activation in neutrophils (Figure 5A). To investigate whether GPR120 regulates neutrophil function through enhanced activation of mTOR, we added rapamycin, an mTOR inhibitor, to the neutrophil cultures with CpdA. Blockade of mTOR suppressed ROS production and NETs formation induced by CpdA (Figures 5B-c).

We then investigated whether GPR120 promotes the neutrophil killing of bacteria through the activation of mTOR. We pre-treated neutrophils with CpdA in the presence or absence of rapamycin and then collected the neutrophils to co-culture with *Citrobacter rodentium*. As shown in Figure 5D, CpdA-treated neutrophils suppressed *Citrobacter rodentium* growth compared with control neutrophils. However, this effect was abrogated in neutrophils treated with CpdA and mTOR inhibitor. Taken together, these data suggest that GPR120 promotes the neutrophil killing of bacteria by neutrophils through the activation of the mTOR pathway.

### Glycolysis mediates GPR120 induction of bacteria-killing by neutrophils

**Figure 6.**
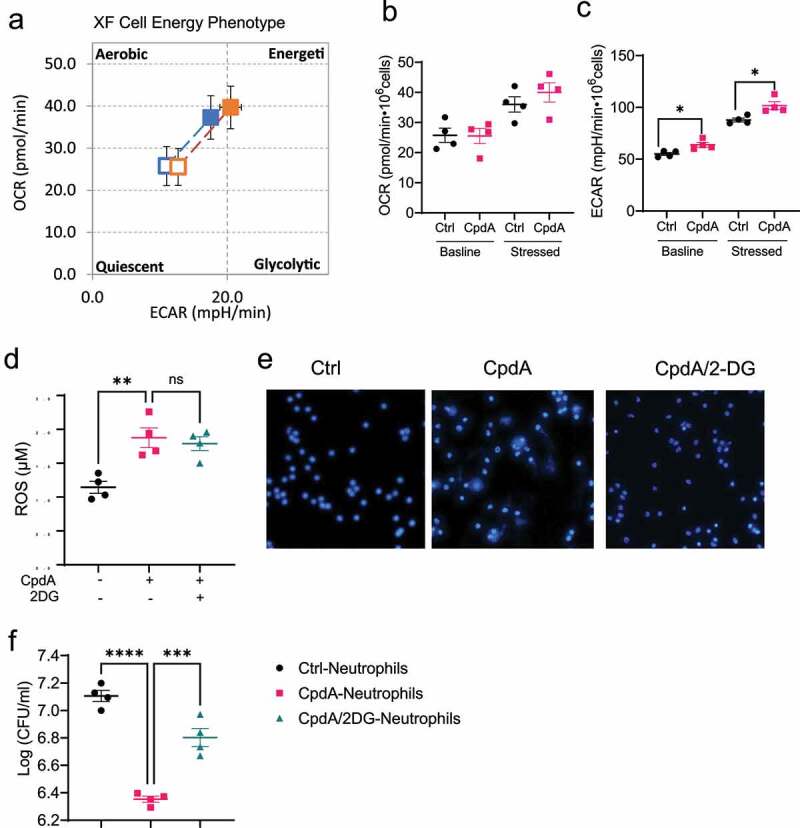
GPR120 regulates NETs formation in neutrophils through the upregulation of glycolysis.

Notes: (**a-c**) Neutrophils (n = 4/group) were treated with or without CpdA (3 µM) for 1 h, and then OCR (**a** and **b**) and ECAR (**a** and **c**) levels were detected by Seahorse XF Cell Energy Phenotype Test using a Seahorse XF96 Analyzer. (**d-e**) Neutrophils were pre-treated with or without CpdA (3 µM) in the presence or absence of 2DG (250 µM) for 1 h. ROS production (**d**) and NETs formation (**e**) was determined. (**f**) Neutrophils were pre-treated with or without CpdA (3 µM) in the presence or absence of 2DG (250 µM) for 1 h, and then co-cultured with *Citrobacter rodentium* in the plates for 12 h. The bacterial suspensions were then transferred to solid MacConkey’s agar culture plates overnight, and CFU was counted. One representative of two independent experiments was shown. Data were expressed as mean ± SEM. Statistical significance was tested by the two-tailed unpaired Student's t-test (**b** and **c**) or the two-tailed one-way ANOVA (**d** and **f**). *p < 0.05, **p < 0.01, ***p < 0.001, ***p < 0.0001.

It has been shown that metabolism is crucial in regulating various functions in different types of cells^26,27^. Next, we investigated whether GPR120 affects glycolysis and mitochondrial oxidation, the two major metabolic events in neutrophils. We treated neutrophils with or without CpdA for 1 h and then measured their energy phenotype using Seahorse XF Cell Energy Phenotype Test Kit. This assay measures the two major energy-producing pathways, mitochondrial respiration and glycolysis, under baseline and stressed conditions, which are stimulated by the ATP synthase inhibitor oligomycin and the mitochondrial uncoupling agent FCCP. The energy phenotype was shown in Figure 6a. Specifically, there was no difference in Oxygen Consumption Rate (OCR), which represents the mitochondrial respiration level, both in baseline and stressed conditions (Figure 6b). However, CpdA-treated neutrophils showed an increased level of Extracellular Acidification Rate (ECAR), which represents the glycolysis levels, compared with control neutrophils under baseline and stressed conditions (Figure 6c). Taken together, GPR120 promotes glycolysis but not mitochondrial respiration under both baseline and stressed conditions.

Next, we determined whether glycolysis is involved in the GPR120 regulation of neutrophil functions. We treated neutrophils with or without CpdA in the presence or absence of 2-Deoxy-D-glucose (2DG), an inhibitor of glycolysis. Treatment with 2DG did not affect ROS production induced by CpdA (Figure 6d) but suppressed GPR120 induction of NETs formation (Figure 6e). Furthermore, the capacity to inhibit *Citrobacter rodentium* growth was decreased in neutrophils pre-treated with CpdA and 2DG compared with neutrophils treated with CpdA alone (Figure 6f). These results indicated that the GPR120 promotes neutrophils killing of bacteria at least partially through upregulation of glycolysis.

### GPR120-activated neutrophils produce higher levels of IL-17A and IL-22 and promote intestinal epithelial cell barrier function

**Figure 7.**
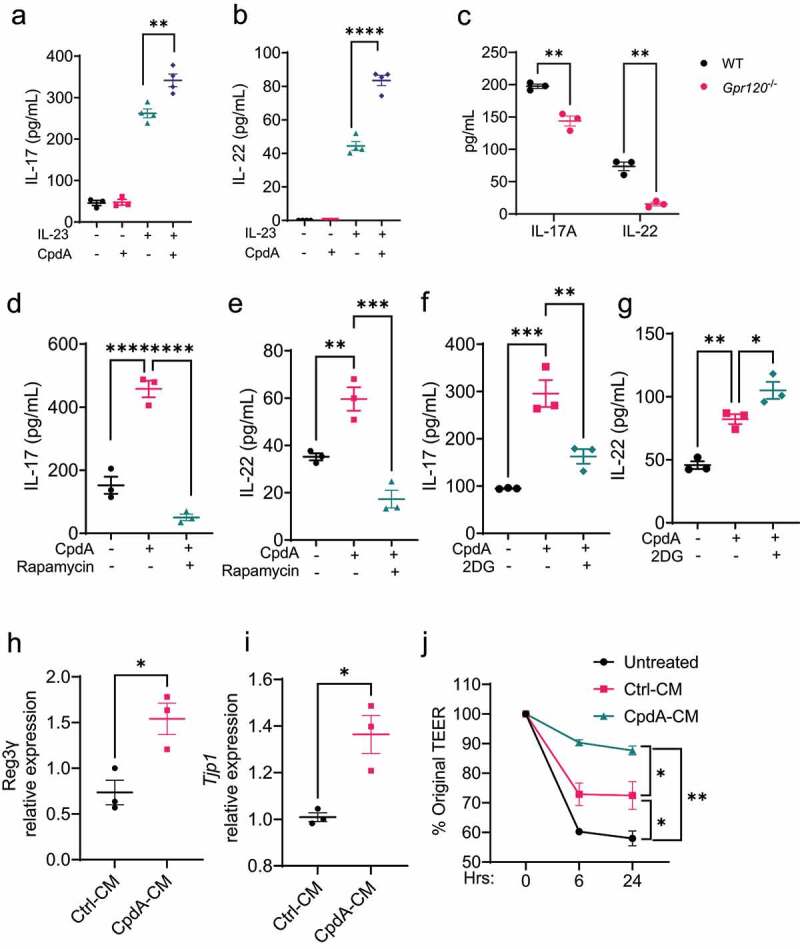
GPR120 regulates IL-17A and IL-22 production and IEC barrier function in neutrophils.

Notes: (**a-b**) WT neutrophils (n = 4/group) were treated with or without CpdA (3 µM) in the presence or absence of IL-23 (20 ng/mL) for 24 h, and IL-17A (**a**) and IL-22 (**b**) production in culture supernatants were measured by ELISA. (**c**) WT and GPR120-deficient neutrophils were treated with IL-23 (20 ng/mL) for 24 h, and IL-17A and IL-22 levels were determined. (**d-g**) Neutrophils (n = 4/group) were treated with IL-23 in the presence or absence of CpdA (3 µM)/rapamycin (2 μM, **d-e**) or 2DG (250 µM, **f-g**) for 24 h, and IL-17 (**d** and **f**) and IL-22 (**e** and **g**) production in culture supernatants were measured by ELISA. One representative of three independent experiments was shown. (**d-i**) Mode-K cells (n = 3/group) were cultured in the medium of control or CpdA-treated neutrophils, and the genes of *Reg3g* and *Tjp1* were determined after 24 h. (J) Mode-K cells (n = 3/group) in the upper inserts were cultured in the medium of control or CpdA-treated neutrophils and treated with proinflammatory cytokines (10 ng/mL of LPS, 40 ng/mL of TNF-α, and 20 ng/mL of IL-1β). TEER levels were determined by Epithelial Volt-Ohm at different time points, and the percentage of original TEER was calculated. One representative of three independent experiments was shown. Data were expressed as mean ± SEM. Statistical significance was tested by the two-tailed unpaired Student's t-test (**a-B**) or the two-tailed one-way ANOVA (**c-F**). **p < 0.01, ***p < 0.001, ***p < 0.0001.

It has been shown that neutrophils produce IL-17A and IL-22, which promote intestinal production of antimicrobial peptides to inhibit bacterial growth and suppress intestinal inflammation^28,29^, as well as TGF-β, which induces intestinal epithelial cell production of amphiregulin to promote intestinal epithelial barrier function^30^. We next investigated whether GPR120 also regulates neutrophil production of IL-17A, IL-22 and TGF-β. We treated neutrophils with or without CpdA in the absence or presence of IL-23, which stimulates IL-17A and IL-22 in neutrophils^31^. As shown in Figures 7a-b, IL-23 promoted IL-17 and IL-22 production, which was further enhanced by CpdA treatment. In addition, GPR120-deficient neutrophils produced significantly lower levels of IL-17A and IL-22 in the presence of IL-23 (Figure 7c). However, CpdA did not affect TGF-β production (Supplementary Figure S5), indicating that GPR120 specifically regulates IL-22 and IL-17 production in neutrophils. In addition, DHA did not affect IL-17A and IL-22 production (Supplementary Figure S3D).

To determine whether mTOR and glycolysis also mediate GPR120 induction of neutrophil production of IL-17A and IL-22, we treated neutrophils with CpdA in the presence or absence of mTOR inhibitor rapamycin or glycolysis inhibitor 2-DG, and measured IL-17A and IL-22 24 h later. As shown in Figures 7d-e, the addition of rapamycin inhibited GPR120 induction of IL-17A and IL-22. However, inhibition of glycolysis decreased IL-17A production but increased IL-22 production induced by CpdA (Figures 7e-f), indicating that glycolysis differentially regulated GPR120 induction of IL-17A and IL-22 in neutrophils.

Considering that IL-17A and IL-22 do not affect bacteria directly, we did not investigate whether CpdA induction of IL-17A and IL-22 contributes to inhibiting bacterial growth in this study. IL-17A and IL-22 have been reported to participate in the anti-bacterial activity by promoting intestinal barrier functions^32,33^. We collected the culture medium from control and CpdA-treated neutrophils and treated the murine IEC cell line, Mode-K, with the conditioned medium. We found that the conditioned medium of CpdA-treated neutrophils induced higher levels of Reg3γ, an antimicrobial peptide, and tight junction protein– (TJP) expression (Figures 7h-i). In addition, the conditioned medium from GPR120-treated neutrophils promoted intestinal integrity upon proinflammatory cytokine insults, as demonstrated by a higher level of transepithelial/transendothelial electrical resistance (TEER) (Figure 7j).

## Discussion

**Figure 8.**
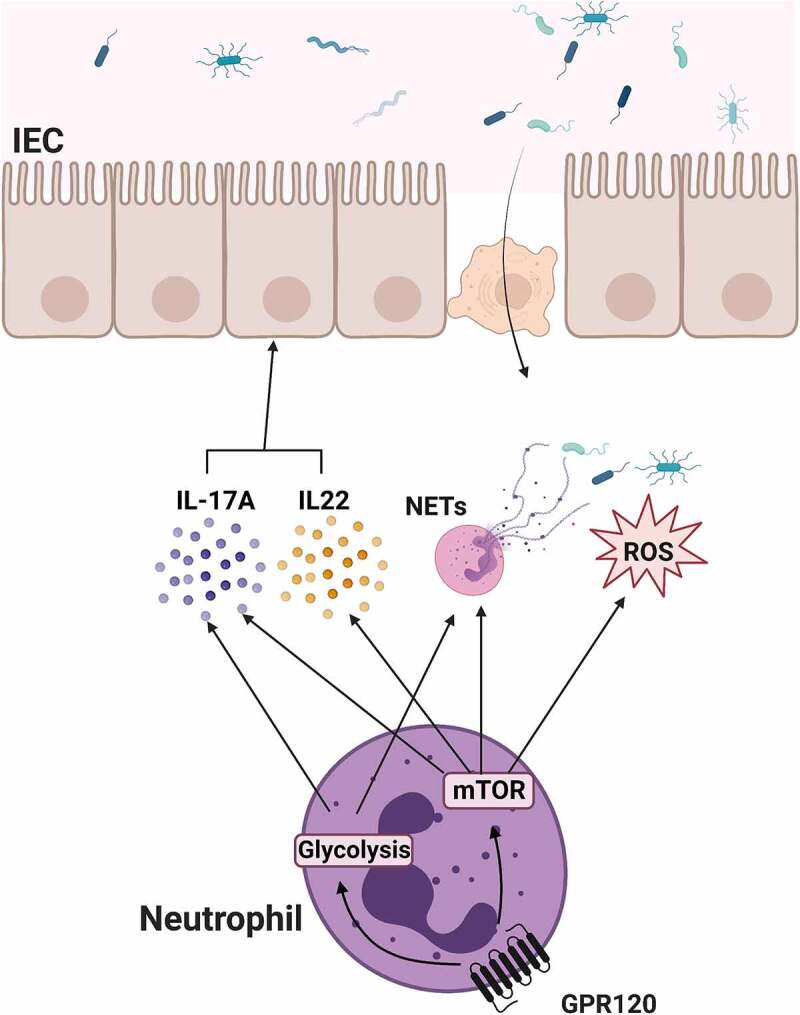
The schematic diagram of GPR120 regulation of neutrophil anti-bacterial function.

Notes: GPR120 activation in neutrophils induces NETs formation and ROS production, which directly enhance anti-bacterial activity. In addition, GPR120 promotes IL-17A and IL-22 production in neutrophils, which contributes to intestinal epithelial barrier integrity. These processes were differentially regulated by enhanced glycolysis and mTOR activation.

Accumulating evidence demonstrates that neutrophils are critical in regulating homeostasis in several tissues and systems, including the intestine. Besides, dietary components also participate in modulating intestinal homeostasis. ω-3 PUFA is commonly consumed from daily diet and has been implicated in the regulation of intestinal microbiota and intestinal disorders. In the current study, we demonstrated that GPR120, a recently recognized receptor for ω-3 PUFA, promotes neutrophil production of ROS and formation of NETs as well as expression of IL-17A and IL-22 via activation of mTOR and upregulation of glycolysis, which contributes to host against bacteria invade (Figure 8). Therefore, our study provides novel insights into how GPR120 contributes to the maintenance of intestinal microbiota homeostasis through the regulation of neutrophil functions.

The gut microbiota has been well recognized in the regulation of intestinal health and diseases, including IBD. Various mechanisms that control the homeostasis of gut microbiota, and different gut bacteria differentially regulate host responses to gut microbiota and pathogens to promote or inhibit intestinal inflammation. It has been reported that *Bacteroidetes* is decreased in IBD patients^16^. *Bacteroidales* S24–7 group, which belongs to *Bacteroidetes* phylum, has been demonstrated to be greatly decreased after the onset of colitis^34^, indicating its role in modulating intestinal inflammation. In this study, we found that the intestinal bacterial load was increased in *Gpr120*^−/−^ mice, and *Gpr120*^−/−^ mice showed different intestinal microbiota profiles, in which the *Bacteroidales* S24–7 group was significantly decreased, suggesting that GPR120 regulates the amounts and composition of gut microbiota, which may favor the maintenance of a healthy gut microbiome.

Neutrophils are recruited to the infection site and then kill bacteria and pathogens mainly by producing ROS and forming of NETs directly^17–20^. Although the effects of GPR120 on other innate cells, including macrophages and dendritic cells, have been investigated^35^, the role of GPR120 in regulating functions in neutrophils is still unknown. In this study, we found that the GPR120 agonist promoted both ROS production and NETs formation in neutrophils. Interestingly, GPR120 agonist enhanced neutrophils to inhibit the growth of enteric bacteria, not only the enteric pathogen *Citrobacter rodentium*, but also gut commensal bacteria *Escherichia coli* O9:H4 and pathobiont *Escherichia coli* O83:H1, which might explain the higher intestinal bacterial load in *Gpr120*^−/−^ mice, indicating that GPR120 contributes to the maintenance of gut microbiota homeostasis probably through regulation of neutrophil controlling of gut bacteria. Furthermore, inhibition of ROS or NETs suppressed neutrophils to kill bacteria induced by GPR120 agonist, indicating that GPR120 promotes neutrophils to inhibit bacteria through upregulation of ROS and formation of NETs. Under healthy conditions, IECs and mucus layer separate commensal bacteria and immune cells, including neutrophils, underneath IECs. Upon injury or colitis or pathogen invasion, commensal bacteria and pathogens could reach IEC and lamina propria. In this case, activation of GPR120 in neutrophils promotes the killing of both commensal bacteria and pathogens when they invade intestinal lamina propria. Although GPR120 promotes neutrophils to kill bacteria, we cannot exclude the possibility of other cells that express GPR120 in changing bacterial composition in *Gpr120*^−/−^ mice. GPR120 affects neutrophil anti-bacterial function, which might contribute, at least partially, to the altered composition of gut microbiota in *Gpr120*^−/−^ mice. Therefore, neutrophil-specific GPR120KO mice is important in exploring the role of neutrophil-expressed GPR120 *in vivo*.

Glycolysis is considered the major metabolism pathway in neutrophils; however, other metabolic pathways, including the pentose phosphate pathway (PPP), the citric acid cycle, and oxidation pathways, have been described recently^36^. In this study, we demonstrated that neutrophils were more glycolytic after the treatment with the GPR120 agonist, whereas the oxidation levels were not altered. Consistent with previous studies showing that glycolysis is involved in NETs formation^37,38^, inhibition of glycolysis suppressed NETs formation induced by GPR120 agonist, which further inhibited neutrophils from killing bacteria. However, the blockade of glycolysis did not affect ROS production, which mainly relies on PPP in neutrophils^12^. Whether PPP is also involved in GPR120 regulation of neutrophils’ anti-bacterial functions was not investigated in this study but should be further studied in the future.

In summary, our study demonstrates that GPR120 promotes neutrophil control of gut bacterial growth. Our study thus provides evidence for GPR120 as a new potential therapeutic target for suppressing intestinal infection and inflammation.

## Materials and methods

### Mice

Wild-type (WT) C57BL/6 mice were obtained from the Jackson Laboratory, and C57BL/6 *Gpr120*^−/−^ mice were obtained from Bristol-Myers Squibb. All the mice were bred and maintained in the animal facilities at the University of Texas Medical Branch. Both male and female mice were used. All experiments were reviewed and approved by the Institutional Animal Care and Use Committees of the University of Texas Medical Branch.

### Reagents

Neutralizing antibody against Ly-6 G (1A8) was purchased from Bio X Cell. Flow cytometry antibodies, FITC- CD11b, PE/Cy7-Ly6G, and Percp/cy5.5-pmTOR, were purchased from Biolegend. Elisa kits were purchased from BioLegend. Mouse recombinant IL-23 was purchased from BioLegend. Culture medium RPMI 1640, DMEM, and HBSS buffer were purchased from Corning. Thioglycolate broth, CpdA, rapamycin, and 2-Deoxy-D-glucose (2DG) were purchased from Sigma-Aldrich. Diphenyleneiodonium (DPI) and hydrochloride (GSK484) were obtained from Cayman. Amplex® red hydrogen peroxide/peroxidase assay kit and Live/Dead Fixable Dead Cell Stain Kit were purchased from Thermo Fisher Scientific. *Citrobacter rodentium* strain DBS100 (ATCC) were obtained from ATCC. *Escherichia coli* O9:H4^39^ and *Escherichia coli* O83:H1^24^ were kindly provided by Dr. Alfredo Torres of UTMB.

### Citrobacter rodentium infection mouse model

WT and *Gpr120*^*-/-*^ mice were orally administrated with *Citrobacter rodentium* (5 × 10^8^/mouse) on day 0. Mice were sacrificed on day 10 post-infection.

For antibody treatment, WT mice were intraperitoneally injected with anti-IgG (4 mg/kg) or anti-Ly6G (4 mg/kg) every day from day 0.

### DSS-induced colitis mouse model

WT and *Gpr120*^*-/-*^ mice were orally treated with 2% DSS (w/v) in drinking water for 7 days, and the water was changed to normal drinking water for another 3 days. Mice were sacrificed on day 10.

### Fecal and splenic Citrobacter rodentium culture

Fresh feces were collected and suspended in cold PBS. After a series of 10-fold dilution, the fecal suspension was seeded onto MacConkey’s agar culture plates. Bacteria CFU counts were normalized to fecal weights. Spleens were immediately collected when mice were sacrificed, homogenized in cold PBS, and seeded onto MacConkey’s agar culture plates. Bacteria counts were normalized to fecal weights. The total bacteria CFU counts in every spleen were determined.

### Ex vivo organ culture

The colons were removed and longitudinally opened. After washing the cold RPMI medium three times, two 3-mm circular full-thickness pieces of the colons were obtained using a 3-mm dermal punch and placed in 1 ml complete RPMI media for 24 h at 37 °C with 5% CO_2_. Culture supernatants from the culture were collected for analysis of cytokine content.

### ELISA

The cytokine production was measured using ELISA kits (IL-17A, IL-22, TGF-β, and TNF-α) according to the manufacturer’s instructions. Ninety-six-well plates were coated with the indicated cytokine capture antibody overnight at 4°C. After blocking using 1% BSA, samples were added to wells and incubated at room temperature for 2 h, followed by incubation with a detection antibody for 1 h. HRP-labeled streptavidin was then incubated for 30 min. After adding tetramethylbenzidine substrate, the absorbance of each well was measured at 450 nm.

### H&E staining and pathological scoring

Colonic tissues were Swiss-rolled and fixed in 10% buffered formalin for 24 h. After dehydration, tissues were embedded, and 5-µm sections were cut. H&E staining was performed after a series of hydration^40^. Images were captured by a Leica microscope. Pathological scores were determined by different key parameters based on different mouse models^41^.

### Quantitative PCR

Bacterial 16S rDNA: Fecal pellets were collected, and fecal DNA was extracted using phenol chloroform. The same amount of DNA was used for measuring 16S counts by quantitative PCR. Primers are the following: 16S forward: 5’-TCCTACGGGAGGCAGCAGT-3’; 16S reverse: 5’-GGACTAC- CAGGGTATCTAATCCTGTT-3’^42^. The data were normalized to eukaryotic β-actin.

Gene expression in Mode-K cells: After treatment, Mode-K cells were collected, and total RNA was extracted by TRizol. 200 ng of RNA was used for reverse transcription, and *Reg3g* and *Tjp1* levels were determined by real-time PCR. Primers are following: *Reg3g* forward: 5’-TCCCAGGCTTATGGCTCCTA-3’; *Reg3g* reverse: 5’-GCAGGCCAGTTCTGCATCA-3’; *Tjp1* forward: 5’- GTTGGTACGGTGCCCTGAAAGA-3’; *Tjp1* reverse: 5’- GCTGACAGGTAGGACAGACGAT-3’. The relative expression was normalized to *Gapdh*.

### 16S rRNA Sequencing

Fecal bacterial DNA was isolated using a QIAAMP PowerFecal DNA kit (Cat# 12830–50, Lot# 160044775, Qiagen) according to the manufacturer’s instructions. The microbiome samples were analyzed using barcoded high-throughput amplicon sequencing of the bacterial 16S rRNA gene. Quality control and taxonomical assignment of the resulted reads were performed using CLC Genomics Workbench 21.0. Microbial Genomics Module (http://www.clcbio.com). Low-quality reads containing nucleotides with a quality threshold below 0.05 (using the modified Richard Mott algorithm), as well as reads with two or more unknown nucleotides, were removed from the analysis. Reference-based OTU picking was performed using the SILVA SSU v132 97% database^43^. Sequences present in more than one copy but not clustered to the database were then placed into de novo OTUs (97% similarity) and aligned against the reference database with 80% similarity threshold to assign the “closest” taxonomical name where possible. Chimeras were removed from the dataset if the absolute crossover cost was 3 using a k-mer size of 6. The beta diversity was estimated using the Bray-Curtis method based on PCoA axes representing the top three highest variances.

### Neutrophil isolation

Mice were injected with 1 ml of 3% thioglycolate broth into the peritoneal cavity. After 4 h, mice were sacrificed, and 10 ml cold PBS buffer containing 5% FBS was injected subsequently intraperitoneally subsequently. After a gentle massage of the abdomen, peritoneal fluid was transferred into centrifuge tubes. Neutrophils were purified using 50% Percoll at 1200 rpm for 20 min.

### Neutrophil culture

Neutrophils were cultured in the RPMI 1640 medium containing penicillin-streptomycin and fetal bovine serum at 37°C and 5% CO_2_ in the presence or absence of CpdA (3 µM) or DHA (5 µM), as well as other inhibitors indicated in the Figure legends. For collecting medium for determining IL-17A and IL-22 production, neutrophils were treated with or without IL-23 (20 ng/mL).

### Viability assay

Resazurin (44 µM) was added to the neutrophil culture medium. Cell viability was calculated by subtracting the absorbance at 595 nm from the absorbance at 570 nm at the time points indicated.

### Reactive oxygen substrate assay

Amplex® red hydrogen peroxide/peroxidase assay kit was used for measuring ROS production secreted by neutrophils. The reaction mixture, which contains 50 µM Amplex® Red reagent and 0.1 U/mL HRP in HBSS, was added into the 96-well plate and pre-warmed at 37°C for 10 min. WT or GPR120-deficient neutrophils were treated with or without CpdA (3 µM), DHA (5 µM), rapamycin (2 µM), or 2DG (250 µM), and then added to the plate. The ROS was determined by the fluorescence for excitation at 560 nm and emission detection at ~590 nm.

### NETs staining

Peritoneal neutrophils were treated with or without CpdA (3 µM), DHA (5 µM), rapamycin (2 µM), or 2DG (250 µM), and then seeded on the poly-lysin-coated coverslips. After 1 h, neutrophils were fixed with 4% paraformaldehyde and stained with Hoechst 33,342 (1 µg/ml) at room temperature for 5 min. The NETs were visualized on a Cytation 5 microscope.

### In vitro bacterial killing by neutrophils

Peritoneal neutrophils were cultured with or without CpdA (3 µM) in the presence of rapamycin (2 µM) or 2DG (250 µM) for 1 h. Neutrophils were collected and co-cultured with appropriate aliquots of *Citrobacter rodentium* strain DBS100 (ATCC), *Escherichia coli* O9:H4, or *Escherichia coli* O83:H1, with an initial OD_600_ value of 0.1–0.2, in the presence or absence of DPI (10 µM) or GSK484 (25uM). The bacterial suspensions were incubated at 37°C for 12 h under aerobic conditions and then transferred to solid MacConkey’s agar culture plates (*Citrobacter rodentium*) or Luria Broth’s agar culture plates (*Escherichia coli* O9:H4 and *Escherichia coli* O83:H1) overnight. Finally, the colony-forming units (CFU) were counted.

### Cell metabolism measurement

Neutrophils were pre-treated with or without CpdA (3 µM). After 30 min of treatment, cells (5 × 10^5^ cells per well) were suspended in Seahorse XF media and seeded into a 96-well Seahorse plate, which was pre-coated with poly-lysin, and subjected to the Seahorse XF Cell Energy Phenotype Assay to determine oxygen consumption rate (OCR) and extracellular acidification rate (ECAR) under baseline and stressed conditions.

### Mode-K cell culture and treatment

Neutrophils were cultured with or without CpdA (3 µM) in the RPMI 1640 medium containing penicillin-streptomycin and fetal bovine serum at 37°C and 5% CO_2_, and the medium was collected after 24 h. Mode-K cells were cultured in 80% DMEM medium containing penicillin-streptomycin, fetal bovine serum, and non-essential amino acid and 20% culture medium of neutrophils at 37°C and 5% CO_2_. Mode-K cells were collected after 24 h for gene expression.

### Transepithelial electrical resistance (TEER) assay

Mode-K cells (300 K) were suspended in 200 µL of DMEM culture medium and then seeded in the insets (0.4 µm polyester membrane). The insets were carefully inserted into the lower chamber (600 µL of DMEM culture medium) of the 24-well Transwell plates. After 24 h, cells were attached to the insert, and the medium in the inserts was changed to a conditioned medium of neutrophils in the presence of proinflammatory cytokines (10 ng/mL of LPS, 40 ng/mL of TNF-α, and 20 ng/mL of IL-1β). TEER levels were determined by Epithelial Volt-Ohm Meter (Millicell ESR-2) at time points indicated.

## Statistical analysis

All the data were analyzed using Prism 9.0 (GraphPad Software, San Diego, CA) and presented as mean ± SEM. Analyses were based on whether the data were normally distributed and the number of tested groups for comparison. *p < 0.05, **p < 0.01, ***p < 0.001, ****p < 0.0001.

## Grant support

This work was supported by NIH grants DK112436, DK125011, AI150210, DK124132.

## Supplementary Material

Supplemental Material

## Data Availability

16S rRNA sequencing data have been deposited in SRA under the BioProject number PRJNA716350 (https://www.ncbi.nlm.nih.gov/bioproject/PRJNA716350).

## Abbreviations

CFU: colony-forming units
DPI: Diphenyleneiodonium
ECAR: Extracellular Acidification Rate
GPR120: G-protein coupled receptor 120
NETs: neutrophil extracellular traps
OCR: Oxygen Consumption Rate
PPP: pentose phosphate pathway
PUFA: polyunsaturated fatty acids
ROS: reactive oxygen species
WT: wild-type
2DG: 2-Deoxy-D-glucose.
