# Correction

**DOI:** 10.1080/19490976.2024.2329383

**Published:** 2024-03-14

**Article title**: GPR120 promotes neutrophil control of intestinal bacterial infection # On a New Line **Authors**: Zheng Zhou, Wenjing Yang, Tianming Yu, Yu Yu, Xiaojing Zhao, Yanbo Yu, Chuncai Gu, Anthony J Bilotta, Suxia Yao, Qihong Zhao, George Golovko, Mingsong Li, and Yingzi Cong

**Journal**: *Gut Microbes*

**DOI**: https://doi.org/10.1080/19490976.2023.2190311

In the original version of this article, the bottom images showing CpdA treatment of Gpr120^−/−^ neutrophils in Fig. 4b were incorrect.

**Figure 4.**
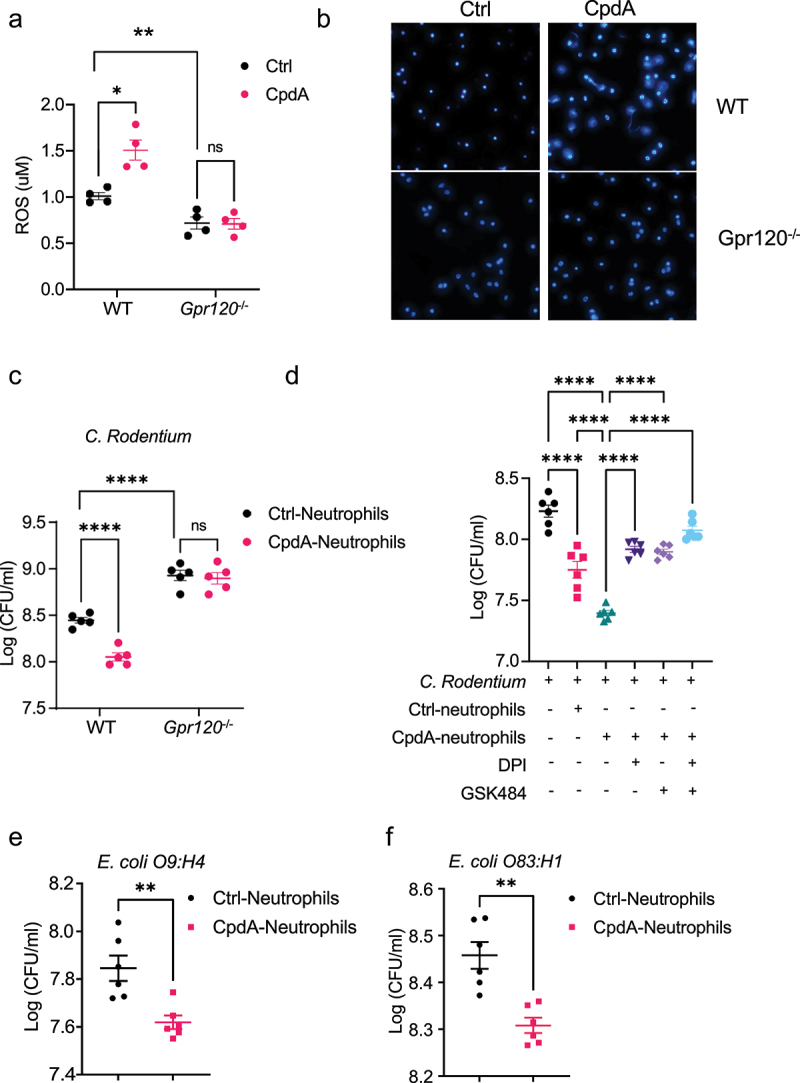
GPR120 agonist promotes neutrophil inhibition of enteric bacterial growth through the upregulation of ROS and NETs. Notes: (**a-b**) WT or GPR120-deficient neutrophils (n = 4/group) were treated with or without CpdA (3 µM) for 1 hour. ROS production was measured using Amplex Red Hydrogen Peroxide Assay Kit (**a**). WT or GPR120-deficient neutrophils were then stained with Hoechst 33342 (blue), and representative NETs were shown (**b**). (**c**) WT or GPR120-deficient neutrophils (n = 5/ group) were pre-treated with or without CpdA (3 µM) for 1 hour, and then co-cultured with *Citrobacter rodentium* in the plates for 12 hours. The bacterial suspensions were then transferred to solid MacConkey’s agar culture plates overnight, and CFU was counted. (**d**) WT neutrophils (n = 6/ group) were pre-treated with or without CpdA (3 µM) for 1 hour and then co-cultured with *Citrobacter rodentium* (or *Citrobacter rodentium* were cultured alone) in the presence of DPI or/and GSK484 in the plates for 12 hours. The bacterial suspensions were then transferred to solid MacConkey’s agar culture plates overnight, and CFU was counted. in the plates for 12 hours. The bacterial suspensions were then transferred to solid MacConkey’s agar culture plates overnight, and CFU was counted. (**e-f**) Neutrophils were pre-treated with or without CpdA (3 µM) for 1 hour, and then co-cultured with *Escherichia coli* O9:H4 (**e**) and *Escherichia coli* O83:H1 (**f**) for 12 hours. The bacterial suspensions were then transferred to Luria Broth’s agar culture plates overnight, and CFU was counted. One representative of three independent experiments was shown. Data were expressed as mean ± SEM. Statistical significance was tested by the two-tailed unpaired Student t-test (**a, c**, and **e-f**) or one-way ANOVA (**d**). **p < 0.01, ***p < 0.001, ***p < 0.0001.

Correct version of Figure 4:

