# Supplementary material for: GPR120 promotes neutrophil control of intestinal bacterial infection
Source: Gut Microbes. 2023 Mar 16;15(1):2190311. doi: 10.1080/19490976.2023.2190311 (PMC10026904; doi:10.1080/19490976.2023.2190311)
Supplement: Supplemental Material [file KGMI_A_2190311_SM5279.pdf]

Supplementary information for

GPR120 Promotes Neutrophil Control of Intestinal Bacterial Infection

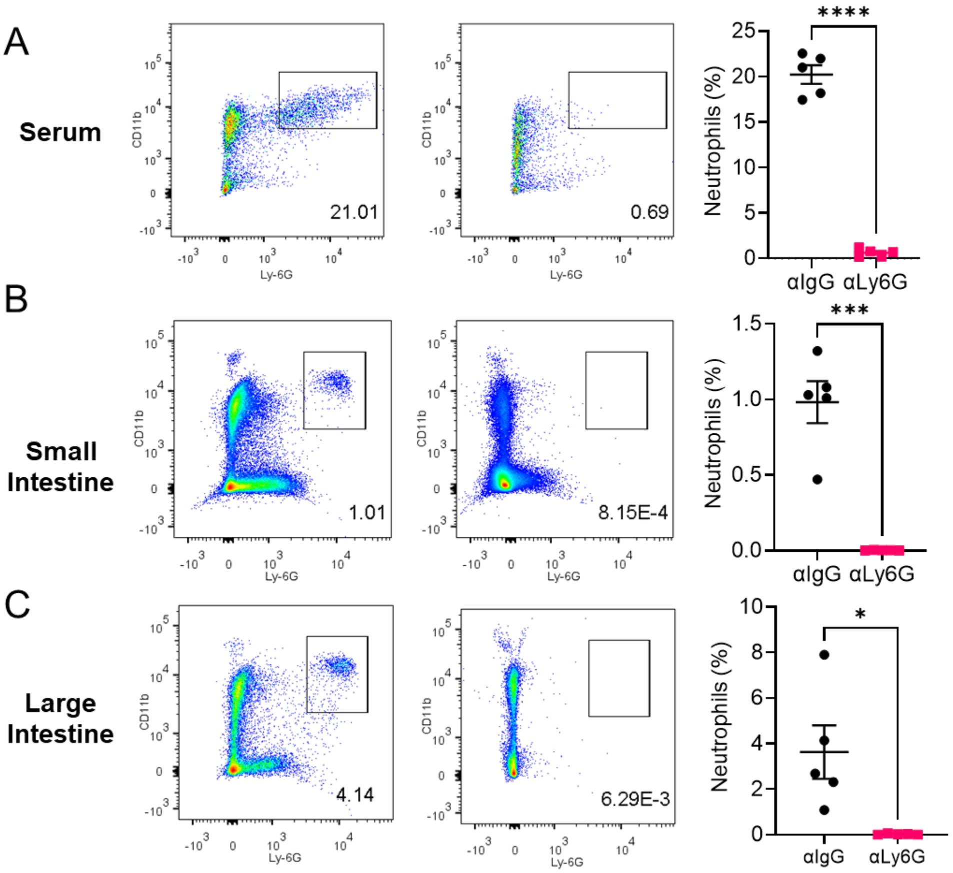

Supplementary Figure 1. The efficiency depletion of neutrophil depletion.

WT mice (n = 5/group) were administered with anti-Ly6G antibody or anti-IgG antibody as control daily. Mice were sacrificed after 10 days. Neutrophils in serum (**A**), small intestine (**B**), and large intestine (**C**) were determined by FACS. Data were expressed as mean ± SEM. Statistical significance was tested by the two-tailed unpaired Student t-test (**A-B**). $^*p < 0.05$, $^{***}p < 0.001$.

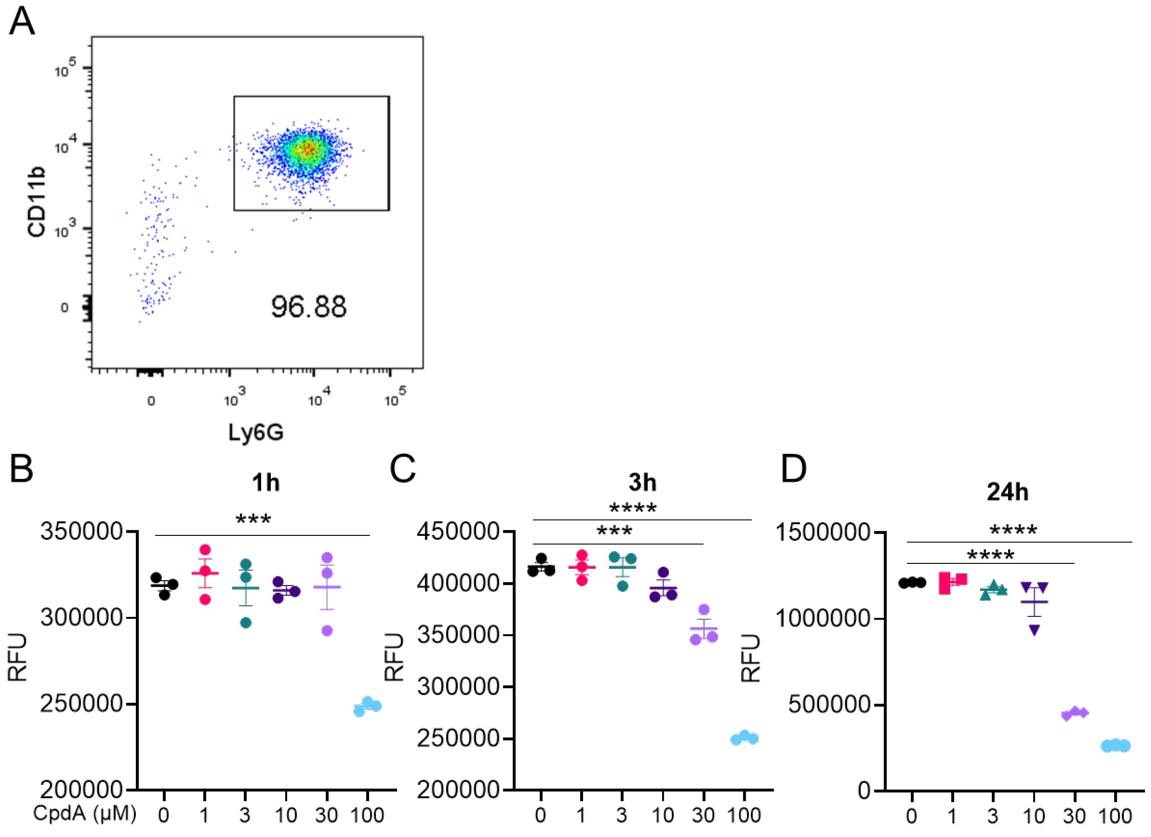

Supplementary Figure 2. The toxicity of CpdA in neutrophils. (**A**) The purity of peritoneal neutrophils was measured by FACS. (**B-D**) The toxicity of CpdA in neutrophils was measured using resazurin viability assay. Relative fluorescence unit (RFU) was measured a fluorometer (Ex=530-570 nm, Em=590-620 nm) 1 hour (**B**), 3 hours (**C**), and 24 hours (**D**) post treatment of indicated doses of CpdA. One representative of two independent experiments was shown. Data were expressed as mean ± SEM. Statistical significance was tested by the two-tailed unpaired Student t-test (**A**) or one-way ANOVA (**B-D**). ***p < 0.001, ***p < 0.0001.

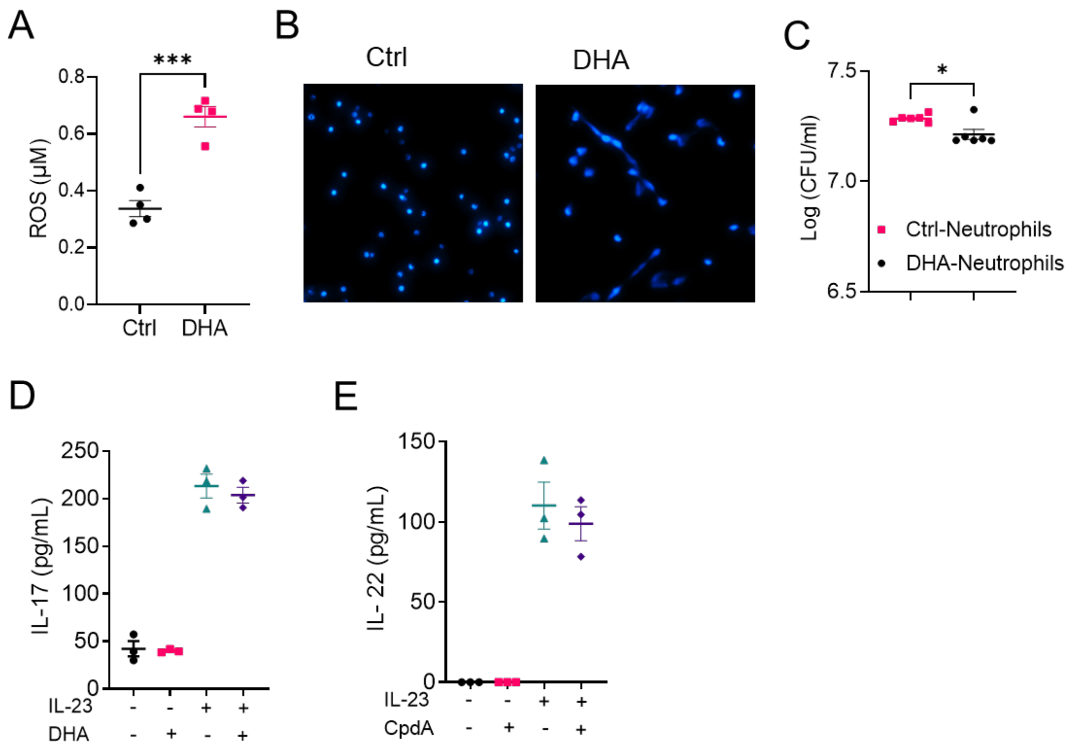

Supplementary Figure 3. DHA enhances neutrophil production of ROS and NET formation, but not IL-17A and IL-22 . WT eutrophils (n = 4/group) were treated with or without DHA (5 μM) for 1 hour. (**A**) ROS production was measured using Amplex Red Hydrogen Peroxide Assay Kit. (**B**) Neutrophils were then stained with Hoechst 33342 (blue) and representative NETs were shown. (**C**) WT neutrophils (n = 6/ group) were pre-treated with or without DHA (5 μM) for 1 hour, and then co-cultured with *Citrobacter rodentium* in the plates for 12 hours. The bacterial suspensions were then transferred to solid MacConkey's agar culture plates overnight, and CFU was counted. (**D-E**) WT neutrophils (n = 4/group) were treated with or without DHA (5 μM) in the presence or absence of IL-23 (20 ng/mL) for 24 hours, and IL-17A (**D**) and IL-22 (**E**) production in culture supernatants were measured by ELISA. One representative of three independent experiments was shown. Data were expressed as mean ± SEM. Statistical significance was tested by the two-tailed unpaired Student t-test (**A, C-E**). *p < 0.05; ***p < 0.001.

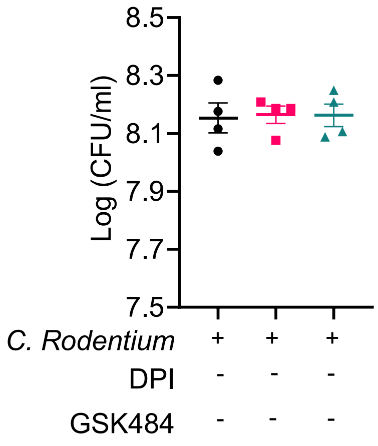

Supplementary Figure 4. DPI and GSK484 does not affect the growth of *Citrobacter rodentium*.

Citrobacter rodentium (n = 4/group) in the presence of DPI or/and GSK484 were cultured in the plates for 12 hours. The bacterial suspensions were then transferred to solid MacConkey's agar culture plates overnight, and CFU was counted. in the plates for 12 hours. The bacterial suspensions were then transferred to solid MacConkey's agar culture plates overnight, and CFU was counted. One representative of two independent experiments was shown. Data were expressed as mean ± SEM. Statistical significance was tested by the one-way ANOVA.

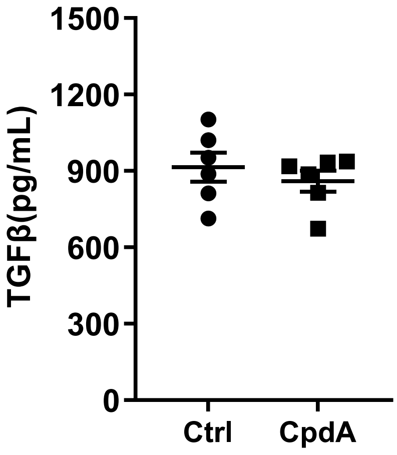

Supplementary Figure 5. GPR120 agonist does not affect TGFβ production in neutrophils. Neutrophils (n = 6/group) were treated with or without CpdA (3 μM) for 24 hours, and TGFβ production in culture supernatants were measured by ELISA. One representative of three independent experiments was shown. Data were expressed as mean ± SEM. Statistical significance was tested by the two-tailed unpaired Student t-test.
